# Financial Risk Protection for Neurosurgical Care in Indonesia and the Philippines: A Primer on Health Financing for the Global Neurosurgeon

**DOI:** 10.3389/fsurg.2021.690851

**Published:** 2021-09-10

**Authors:** Kevin Paul Ferraris, Maria Eufemia C. Yap, Maria Cristina G. Bautista, Dewa Putu Wisnu Wardhana, Sri Maliawan, I Made Ady Wirawan, Rohadi Muhammad Rosyidi, Kenny Seng, Joseph Erroll Navarro

**Affiliations:** ^1^Section of Neurosurgery, Department of Surgery, Jose R. Reyes Memorial Medical Center, Manila, Philippines; ^2^Department of Surgery, Las Piñas General Hospital and Satellite Trauma Center, Las Piñas, Philippines; ^3^ThinkWell, Manila, Philippines; ^4^Department of Economics, Finance and Accounting, Graduate School of Business, Ateneo de Manila University, Makati, Philippines; ^5^Faculty of Medicine, Division of Neurosurgery, Department of Surgery, Udayana University Hospital, Udayana University, Bali, Indonesia; ^6^Faculty of Medicine, Division of Neurosurgery, Department of Surgery, Sanglah General Hospital, Udayana University, Bali, Indonesia; ^7^Faculty of Medicine, Department of Public Health, Udayana University, Bali, Indonesia; ^8^Faculty of Medicine, Department of Neurosurgery, West Nusa Tenggara Province Hospital, Mataram University, Mataram, Indonesia; ^9^Division of Neurosurgery, Department of Neurosciences, University of the Philippines–Philippine General Hospital, University of the Philippines College of Medicine, Manila, Philippines

**Keywords:** health financing, global neurosurgery, social health insurance, strategic purchasing, conditional cash transfer (CCT), out-of-pocket (OOP) expenses

## Abstract

Which conditions treated by neurosurgeons cause the worst economic hardship in low middle-income in countries? How can public health financing be responsive to the inequities in the delivery of neurosurgical care? This review article frames the objectives of equity, quality, and efficiency in health financing to the goals of global neurosurgery. In order to glean provider perspectives on the affordability of neurosurgical care in low-resource settings, we did a survey of neurosurgeons from Indonesia and the Philippines and identified that the care of socioeconomically disadvantaged patients with malignant intracranial tumors were found to incur the highest out-of-pocket expenses. Additionally, the surveyed neurosurgeons also observed that treatment of traumatic brain injury may have to require greater financial subsidies. It is therefore imperative to frame health financing alongside the goals of equity, efficiency, and quality of neurosurgical care for the impoverished. Using principles and perspectives from managerial economics and public health, we conceptualize an implementation framework that addresses both the supply and demand sides of healthcare provision as applied to neurosurgery. For the supply side, strategic purchasing enables a systematic and contractual management of payment arrangements that provide performance-based economic incentives for providers. For the demand side, conditional cash transfers similarly leverages on financial incentives on the part of patients to reward certain health-seeking behaviors that significantly influence clinical outcomes. These health financing strategies are formulated in order to ultimately build neurosurgical capacity in LMICs, improve access to care for patients, and ensure financial risk protection.

## Introduction

The costs accompanying the provision of neurosurgical care in low middle-income countries (LMICs) constitute as barriers to access among the socioeconomically disadvantaged (1). For the often life-threatening neurosurgical conditions, financial barriers that need to be overcome frequently cause economic hardships to the household in which a patient belongs to. Families with a patient needing neurosurgical care are at risk for financial catastrophe and impoverishment, and this is especially true in LMICs (2, 3). The increasing costs of healthcare—including neurosurgery—also further burdens the existing health financing mechanisms in the health systems of LMICs. Particularly in low-resource settings of public hospitals where the poorer segments of the population are served, financial risk protection becomes an important consideration (4, 5). The elimination of catastrophic and impoverishing expenditures remains an important goal for health systems to improve the accessibility of neurosurgery. In this article, we explore the most financially catastrophic neurosurgical disorders that are encountered in public neurosurgical centers in Indonesia and the Philippines. We also describe public-health perspectives that may provide insight for potential solutions.

## Materials and Methods

### Cross-Sectional Survey

The primary aim of the survey was to determine the most commonly encountered neurosurgical conditions that result in financial catastrophe. A secure online survey tool (Google Forms®) was used to disseminate the questionnaire to neurosurgeons currently practicing at public hospitals in Indonesia and the Philippines. Respondents comprised a purposive sample of neurosurgeons invited through the electronic mailing lists of neurosurgical training programs, email to personal contacts, and social media platforms (WhatsApp and Viber). The survey was open between 16th February 2021 and 16th March 2021. Those eligible to complete the survey were neurosurgeons—either as residents or as consultants—whose practice involves a publicly-funded neurosurgical center as part-time or full-time healthcare provider. Duplicates were removed via matching those of an identical name and hospital. Because no patient information was requested from the survey, approval by an institutional review board was not needed. It is of note that while the vantage point of patients would be most ideal to take, the survey has the limitation of inquiring only from the perspective of neurosurgeons as health service providers because of its cursory nature in bringing the more important discussion on solutions to the fore.

### Application of Public Health Perspectives

Using principles and perspectives from public health, contractual management, and financial management, we proffer potential solutions that can address the gaps in health financing of neurosurgical services in Indonesia and the Philippines. When viewed in a public-health context, the problem of catastrophic and impoverishing expenditures becomes more manageable. Existing frameworks from these disciplines permit exploration of financial risks that burden the patients that can potentially be averted by the roles of various agents in the surgical and health systems. We propose a conceptual framework for implementation to inform future policymaking related to the financing of public neurosurgical services in Indonesia and the Philippines.

## Results and Discussion

### Survey Results

A total of 43 neurosurgeons−29 (67%) from Indonesia and 14 (33%) from the Philippines—had valid responses to the survey. Majority of respondents (31.8%) cited malignant intracranial tumors as the neurosurgical condition that causes the worst economic hardship among patients and their families, followed by traumatic brain injury (27.3%), spinal trauma and spinal cord injury (15.9%), and aneurysmal subarachnoid hemorrhage (15.9%).

**Figure 1:**
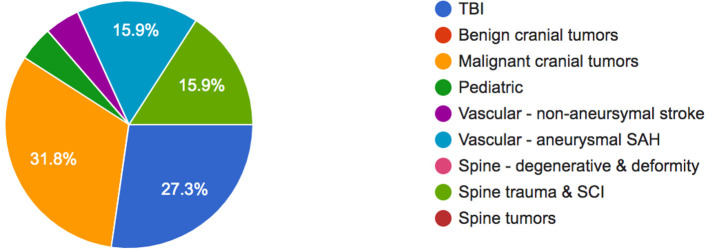
Neurosurgical conditions causing the worst economic hardship among patients.

In terms of out-of-pocket (OOP) expenses for these conditions, malignant intracranial tumors were reported to impose the highest OOP expenses (43.2%), followed by emergency vascular diseases (38.6%), traumatic brain injury (34.1%), and degenerative spine disorders (34.1%) (Figure 1).

**Figure 2:**
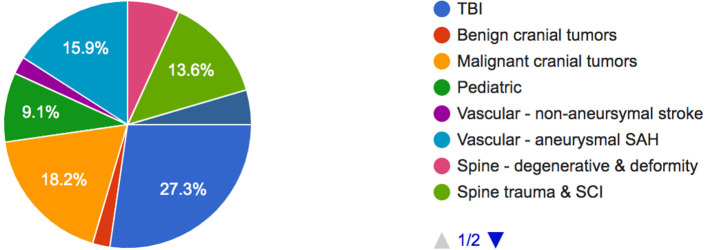
Neurosurgical conditions necessitating greater financial subsidy.

Among the neurosurgical conditions needing greater financial subsidy, traumatic brain injury (27.3%) needed the most, followed by malignant intracranial tumors (18.2%), aneurysmal subarachnoid hemorrhage (15.9%), and spinal trauma and spinal cord injury (13.6%) (Figure 2).

### Socioeconomic Context of Neurosurgical Care

Few studies have examined the association between patient-level socioeconomic factors and outcomes across various diseases that are treated by the specialty of neurosurgery—pediatric hydrocephalus (6–8), craniosynostosis (9), intracranial tumors (10–13), aneurysmal subarachnoid hemorrhage (14, 15) and stroke (16, 17), traumatic brain injury (18–21), spine disorders (22) and spinal cord injury (23, 24). Many of these studies describe the more frequently unfavorable outcomes among the inadequately insured and those with lower socioeconomic status. Due to the inherently costly nature of neurosurgical interventions, many of the socioeconomically disadvantaged patients seek the necessary care from public hospitals (4, 5). Whereas such public neurosurgical centers in Indonesia and the Philippines are mandated by legislation and government regulation to provide healthcare at minimum or zero cost, the reality is that many patients still incur out-of-pocket expenses (25, 26). These expenses are often catastrophic and push their families into impoverishment. Financial catastrophe from accessing surgery remains high in LMICs, and most of those exposed to such financial risk live in sub-Saharan Africa and Southeast Asia (1). On the basis of the dearth of literature on global surgery research related to health financing (27), we explore the contextual solutions for the costly care of socioeconomically disadvantaged patients suffering from malignant intracranial tumors and traumatic brain injury in Indonesia and the Philippines.

### Supply and Demand Sides of Neurosurgical Care

There are various reasons for the still prevalent high out-of-pocket expenses in LMICs, particularly in Indonesia and the Philippines. From the demand side of the healthcare system, both medical and non-medical costs account for the financial catastrophe suffered by poor patients following surgery (2). Surgical conditions result in a greater household poverty effect relative to other health problems and diseases (1, 2, 28). Poverty plays a role in the unaffordability of transportation costs and caregiving expenses, while prolonged hospitalization results in income regression, unemployment, and opportunity costs for both the patient and the caregiver. The sociocultural disadvantage of lower levels of education among the poorer patients contributes to the more advanced presentation of disease at the time of first consultation. Inadequate health insurance also contributes to hesitancy in timely health-seeking behavior—a problem that poses a disadvantage in outcomes after treatment and prognosis. Furthermore, it does not help that any neurosurgical intervention, or any surgical treatment for that matter, is perceived to be costly and expensive (1). Globally, LMICs have the highest rates of catastrophic expenditures following receipt of surgical care; and within any country, most of the burden falls on the poorer segments of the population (2).

From the supply side of the health system—the healthcare providers—publicly-funded hospitals generally provide the needed wide range of emergency and elective neurosurgical care for poor patients. These hospitals are primarily financed from taxation and are appropriated a yearly budget from the national government through the Department or Ministry of Health, or from local or state-run universities. Many public hospitals do not have the bare minimum neurosurgical implements, surgical intensive care units, and even a computed tomography scanner. These complicate the usual treatment pathway for a neurosurgical patient—turning it into a cumbersome navigation around a generally disjointed referral network of hospitals (4, 5, 29). Despite the finite resources that beset government-financed hospitals, neurosurgical care provision in most centers has been largely sustained by the presence of an academic neurosurgical training program. The few hospitals with neurosurgical residency programs in Indonesia and the Philippines are generally better equipped to handle the more complicated and full range of neurosurgical diseases and conditions (30). And yet, like many of the public hospitals in low-resource settings, the efficiency of care is hampered by bureaucratic processes and protocols (1). Commonplace problems like the breakdown of equipment can take time to repair. The purchase of new equipment often gets encumbered in elaborate procurement processes (31). In these respects, the financing of equipment purchase and patient management can have an important impact on the quality and efficiency of neurosurgical care.

### Health Financing: An Important Health Systems Function

Broadly, health financing comprises the mechanisms by which money is mobilized to fund and pay for health-sector activities, and how it is used, raised, and paid out to achieve health-related outputs (32). Financing and payment arrangements determine how much money is available or paid out, who bears the financial burden, who controls the funds, whether healthcare costs are controlled, and which groups or health-system actors are given the incentive (33). A chief method of health financing is by way of health insurance. The organization and pooling of both funds and risks are foremost functions of a health insurance scheme. Many countries, including both Indonesia and the Philippines, have some form of social health insurance that is overseen or managed by the government. Health financing, in a pragmatic sense, is also a matter of policy. These policies and regulations create powerful incentives that influence the actions of individuals and organizations in a given healthcare system.

Socioeconomically disadvantaged patients who seek healthcare services are often price-sensitive because their limited means do not allow them to have the capacity to economically withstand adverse life events such as having a neurosurgical illness requiring hospitalization. In countries that do not have a fully responsive health financing or social protection mechanism—which is more often the case in LMICs—poor households with a sick member would finance their health-related costs by way of out-of-pocket expenses (1, 2). The out-of-pocket expenses they bear can become “catastrophic,” i.e., household spending equal or above 40% of non-subsistence income (1, 2). Incurring health-related catastrophic expenditures pushes patients' families into impoverishment and affects the volume and timeliness of healthcare used, in turn influencing overall health outcomes (32). As far as healthcare utilization is concerned, the extent of social health insurance coverage in public hospitals is therefore important. Universal health coverage and a high degree of responsiveness in curbing out-of-pocket expenses on the part of the social health insurance can improve the accessibility of surgical services, including that of neurosurgery.

### Expanded Functions for Social Health Insurance

**Figure 3:**
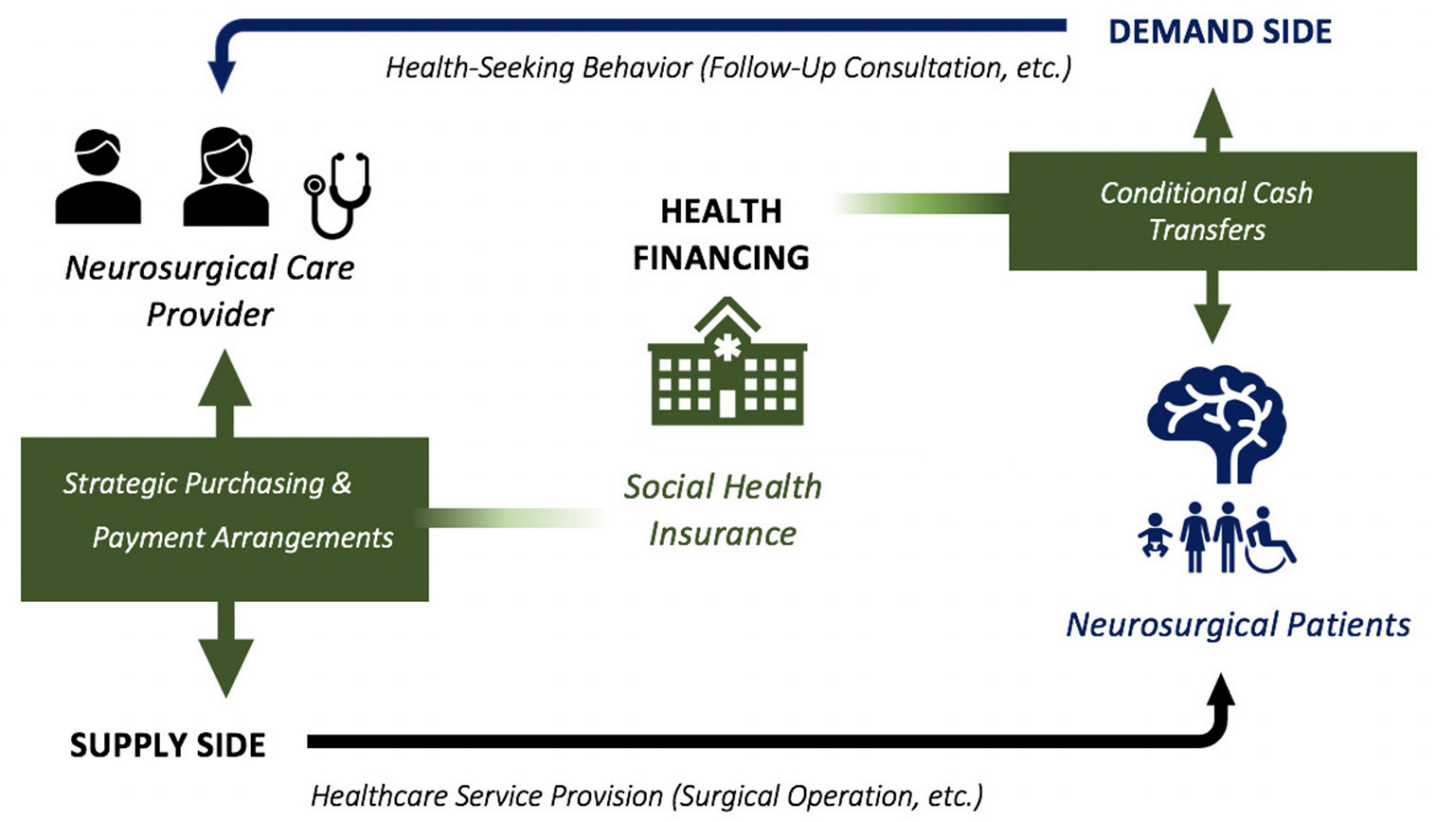
Framework for policy and implementation of health financing as applied to neurosurgery.

Social health insurance (SHI) at the national level acts as a payer for health care goods and services in a country. The Badan Penyelenggara Jaminan Sosial Kesehatan (BPJSK) is the single SHI agency in Indonesia while its counterpart is the Philippine Health Insurance Corporation (PHIC) in the Philippines (26, 34). The BPJSK and the PHIC finance essential primary care and specialized care of majority of the acute and chronic illnesses throughout both the private and public hospital networks. In the face of limited resources in the public health sectors of Indonesia and the Philippines, SHI agencies have to do “strategic purchasing” —essentially to be cost-conscious and efficient in the selection of cases to be paid for from the pooled funds. Financial incentives or other mechanisms can motivate surgical providers to improve quality and efficiency of care, or to respond to increased patient demand. Similarly, poor patients respond to cost savings in the treatment of their illnesses. SHI agencies can certainly play a role in providing economic incentives—in the form of conditional cash transfers, for example—for improving intermediate indicators of treatment success as well as clinically important outcomes. See Figure 3 above for the framework.

## Potential Health Financing Solutions

### Supply-Side Financing: Strategic Purchasing of Neurosurgical Services

Ideally, the role of the SHI is to ensure that adequate resources are mobilized to meet the service entitlements of insured patients—ideally the whole population—and thus achieve “universal health coverage.” While financial risk pooling is important for equity considerations and financial risk protection of the insured, an added function of strategic purchasing by the SHI can drive the quality and efficiency of healthcare provision (1, 32, 35). Certain strategic purchasing methods can modify healthcare providers' behaviors in their treatment decisions and ultimately influence the quantity, quality, and efficiency of neurosurgical care for example. The choice of which disease condition to subsidize is a matter of policy and executive decision, but is mostly founded on data on the costs of treatment, the burden of the disease, and the cost-effectiveness of interventions (36). Assuming the role of strategic purchaser, the SHI selectively enters into a contract with neurosurgical care providers. Providers in this sense would mean the hospital or institution including the neurosurgeons and allied health professionals. The purchaser and the provider then agree on a provider payment method for a standard treatment regimen.

Consider for example—traumatic brain injury (TBI)—for which the current standard of neurosurgical management is entrenched in universally accepted clinical practice guidelines (37). Prehospital management of a brain-injured patient is time-critical. In an analysis of the largest TBI database—the International Mission for Prognosis And Clinical Trial (IMPACT) database—Mushkudiani and colleagues concluded that outcome following TBI is also dependent on race and to a lesser extent on the level of education of the patient, even when adjusting for other causes (18). The underlying reason can be traced to limited access to acute and post-acute care (20, 21); therefore, prehospital and acute hospital care systems ought to be efficiently responsive to socioeconomically disadvantaged patients with TBI (38). Indonesia and the Philippines have the twin problem of a low neurosurgeon-to-population ratio −1 per 731,000 and 1 per 807,000, respectively (30)—and a high prevalence of TBI estimated at around 930 per 100,000 population in the Southeast Asia/Western Pacific region (39). This situation is exacerbated by constraining financial considerations whereby a poor TBI patient often gets rushed to the emergency department of a much farther public hospital despite the proximity of a private hospital with neurosurgical services at the time of injury. This adverse selection by private hospitals presents an added disadvantage for an already economically disadvantaged patient with TBI, whose clinical outcome could have been better were it not for a delayed neurosurgical treatment.

Conceptually, strategic purchasing allows buying of health services for certain groups of patients and uses financial levers and payment schemes that influence the behavior of providers including hospitals and neurosurgeons (35, 36, 40). The results of our survey in this study indicate that TBI is viewed as requiring greater financial subsidies, as similarly found in other studies (22, 23, 41). The case for subsidizing the full costs of treatment for patients with TBI is also justified by its cost-effectiveness (42). Strategic purchasing can realize this through contractual arrangements that would link attractive payment mechanisms to the goal of broadened access to emergency neurosurgical care for TBI patients who are brought to the nearest emergency care facility without regard to the patient's ability to pay or the type of facility, whether public or private. Given the context of a low neurosurgical workforce in both Indonesia and the Philippines, a task sharing scheme with colleagues from the fields of trauma and general surgery can also be contractually set in place with the goal of increasing access to timely neurosurgical trauma referral (43). A catastrophic illness benefit package may be formulated by both the BPJSK and the PHIC that would elevate the financial incentive for all neurosurgical care providers to accept and manage TBI patients regardless of socioeconomic status (44, 45). Greater acceptability and buy-in of this mechanism by neurosurgical providers are afforded by strategic purchasing because the costing and payment schedules are negotiated and prearranged in collaboration with the providers themselves. The payments from SHIs to neurosurgical providers can be further complemented by an element of pay-for-performance (32), with a proportion of the fixed payment withheld and paid according to certain indicators such as timeliness of the surgical operation (46), long-term outcomes assessment (47), and even patient satisfaction with the neurosurgeon-patient engagement (48). In this way, the goal of quality care can be achieved for the neurosurgical management of patients with TBI.

A prospective payment scheme brought about by strategic purchasing can enable the pooling of paid out funds on the part of provider hospitals to accrue future savings (36). The increased generation of revenue at the level of the hospitals can result in upfront investments that will finance the capital outlay costs for scaling up infrastructure and equipment needs, e.g., expansion of neurosurgical bed capacity, addition of dedicated neurosurgical operating theaters, or procurement of costly neurosurgical equipment. In this manner, the improved capacity of the provider hospital can increase neurosurgical volume and the goal of increased access to neurosurgical care can be achieved. In Table 1 below, we summarize other examples of strategic purchaser-provider arrangements.

**Table 1:** Purchaser-provider contractual management for health financing.

Strategic purchasing functions^*^	**Examples for neurosurgery**
1 Providing an appropriate range of services and locations relative to the distribution of the population by means of effective gatekeeping and referral	•Assignment of tiered neurosurgical centers (e.g., higher level and lower level) with a predefined catchment population based on geography and complexity of disease condition •Creation of centers of excellence for certain neurosurgical disease conditions based on available expertise of subspecialist neurosurgeons
2 Selecting providers for accreditation	•Designating and credentialing private hospitals that are capable of accepting and managing traumatic brain injury and emergency neurovascular diseases •Selective contracting and progressive policy of deliberated reimbursement of expenses by accredited private hospitals that are incurred during emergency operations for indigent patients with traumatic brain injury and stroke
3 Implementing provider payment methods efficiently, as in a prospective payment system	•Creation of a catastrophic illness benefit package for traumatic brain injury and/or malignant intracranial tumors •Capitation funding for every indigent patient who gets admitted in a private hospital on an emergency basis and who needs life-saving neurosurgical intervention
4 Making use of monopsonistic purchasing power	•Public-private partnership and debt financing for the capital outlay of infrastructure projects •Economies of scale purchasing arrangements for neurosurgical consumable implements
5 Introducing generic essential drugs, devices, or implants lists	•Creation of a medicines access program for disease-altering drugs, e.g., temozolamide for patients with gliomas •Consignment and purchasing arrangements for steady supply of cranial and spinal implants and instrumentation
6 Monitoring provider performance in terms of quality and efficiency	•Establishing a prospective database of outcomes report following surgery and adjuvant chemoradiotherapy for patients with gliomas •Merit-based payment and reimbursement of neurosurgical centers based on volume-outcome relationships, e.g., high-volume centers doing >10 intracranial aneurysms per year will have seamless financing for purchase of aneurysm clips
7 Ensuring mutual accountability between purchasers and providers through timely payments to healthcare providers and appropriate audit systems	•Checks and balances system that would minimize fraud and pilferage of payments and reimbursements, e.g., repeated neuroimaging of neurosurgical patients will have to be easily cross-checked through linkage with information technology systems •Strict penalty system for breach in contracts when it comes to timeliness of reimbursements, especially for time-critical and life-threatening neurosurgical operations

* *Adapted from: Trisnantoro L, Hendrartini J, Susilowati T, Miranti PAD, Aristianti V. Chapter 3: A critical analysis of selected healthcare purchasing mechanisms in Indonesia. In: Honda A, McIntyre D, Hanson K, Tangcharoensathien V. ed. by. Strategic Purchasing in China, Indonesia, and the Philippines (Comparative Country Studies, Vol. 2 No. 1 2016). Geneva: World Health Organization; 2016. p. 124*.

### Demand-Side Financing: Conditional Cash Transfers for Neurosurgical Patients

Neurosurgical patients, herein considered the demand side of healthcare provision, constitute an important stakeholder in terms of health financing. Despite the fact that surgery in public hospitals is completely subsidized by the BPJSK in Indonesia and the PHIC in the Philippines—at little to zero cost for indigent patients—there remain substantial financial barriers (36). Frequently, financial catastrophe comes from non-medical costs of care, such as the cost of transportation and living expenses of the caregiver (1, 2). For socioeconomically disadvantaged patients in the Philippines, even in-person outpatient follow-up after neurosurgery entails catastrophic expenses and may cause adverse life events that can negatively impact health-seeking behavior overall (49).

The results of our survey show that out-of-pocket expenses surrounding the management of those with malignant intracranial tumors are a prevalent form of health financing in Indonesia and the Philippines. For majority of patients with brain cancer in Indonesia and the Philippines, the standard Stupp protocol for the treatment of glioblastoma could bankrupt their families—often resulting in the patient never receiving or completing the disease-altering adjuvant chemotherapy and radiotherapy. Adjuvant chemotherapy for glioblastoma is carried out on an outpatient basis: consultations are done at the oncology clinic during the course of the patient's ongoing intake of oral drugs (temozolamide). However, SHIs are currently not fully responsive in the aspect of financing for adjuvant therapy and outpatient care. The responsibility of payment for the adjuvant treatments is frequently left to the social welfare division of the hospital or external charitable organizations (45). In the Philippines, the PHIC pays for a so-called “Z Benefit” package for the full treatment course of certain catastrophic illnesses including early breast, colon, and rectum cancers, but not brain cancer. The subsidies offered by the PHIC for colorectal and breast cancer patients have helped achieve better mortality rates and overall better compliance to treatment. In Indonesia, patients with glioblastoma get full subsidies but the BPJSK no longer routinely pays for the treatment costs of recurrent glioblastoma. In the two countries, this situation results in dismal follow-up at baseline and at worst, poor long-term survival among patients with glioblastoma.

The case for subsidizing the full costs of treatment for patients with glioblastoma is justified by its cost-effectiveness (50) and the fact that brain cancer is one of the leading neurological causes of mortality in the Southeast Asian region that are relevant to the neurosurgeon (51). While full subsidies for the Stupp protocol through some form of strategic purchasing appears to be the preeminent solution, the added strategy of using cash transfers for patients with glioblastoma can modify the behavior of the demand-side of neurosurgical care provision. Conditional cash transfers (CCT)—in which recipients are given some amount of money, often conditional on their adherence to a desired behavior—have been used by social welfare programs to reduce poverty and to incentivize salutary action for a number of public-health interventions (52, 53). In Indonesia and the Philippines, a CCT scheme is employed in both countries as a form of social assistance; but unique to the Philippine context is the use of CCT to encourage health-related behaviors among household beneficiaries in addition to the requirement of sending the children to school (54). This health-related requirement however, is only limited to attendance in family development lectures on parental, family, and community responsibilities (54). While there is high-quality evidence that the effects of CCT translate to better outcomes in terms of family and child health (55), similar evidence for surgical, or even neurosurgical care for that matter, currently remains limited. Determining the optimal amount of cash transfers that would modify the health-seeking behavior of surgical patients can be difficult but recent findings from a modeling study suggest a dose-response relationship between the amount of money given and compliance to scheduled surgery (56). The study by Strader et al. noted that until after baseline costs of surgery are paid for by the purchaser—and thus becomes free for the patient—only then can any incremental increase in the amount of cash transfer be expected to improve compliance.

CCTs for patients with glioblastoma may be done to decrease the no-show rate in follow-up consultation schedules and improve the all too common problem of loss to follow-up. The direct provision of cash for indigent patients can be made contingent on their adherence to adjuvant treatment following neurosurgical operation for glioblastoma. In turn, the families' non-medical costs are also subsidized. Furthermore, CCTs also have positive externalities and can be expected to increase the propensity of the household members to have some degree of trust in the systems of healthcare provision and health financing. In this manner, financial barriers to neurosurgical services are actually lowered and a system is set in place to reward beneficial health-seeking behavior that impacts patient outcomes.

## Conclusion

Neurosurgical patients living in Indonesia and the Philippines who are socioeconomically disadvantaged to begin with have the added challenges of incurring out-of-pocket expenses and risking financial catastrophe whenever they navigate the health system for treatment. This situation calls for a more robust and responsive health financing for neurosurgical services. From an economic perspective, health financing for neurosurgery entails the proposition for addressing the demand (patient) or supply (provider) sides of healthcare provision. Strategic purchasing enables a systematic and contractual management of payment arrangements that provide performance-based economic incentives for providers that can in turn help achieve the goals of quality and efficiency of neurosurgical care provision. Conditional cash transfers similarly leverages on financial incentives on the part of patients to reward certain health-seeking behaviors that significantly influence clinical outcomes. These health financing strategies are formulated in order to ultimately build neurosurgical capacity in LMICs, improve access to care for patients, and ensure financial risk protection. It is hoped that this article provides a framework for implementation and affords a basic understanding of health financing that can inform policy and advocacy work among neurosurgeons globally.

## Data Availability Statement

The original contributions presented in the study are included in the article/supplementary material, further inquiries can be directed to the corresponding author/s.

## Author Contributions

KF, MY, and MB initiated and conceptualized the article design. KF drafted the manuscript. KF and DW collected and interpreted the data. MY, MB, SM, IW, RR, KS, and JN critically examined and commented on the manuscript. All authors revised and approved the manuscript.

## Author Disclaimer

The opinions expressed by the authors in the discussion of this article do not necessarily reflect the opinions of the institutions to which the authors belong.

## Conflict of Interest

The authors declare that the research was conducted in the absence of any commercial or financial relationships that could be construed as a potential conflict of interest.

## Publisher's Note

All claims expressed in this article are solely those of the authors and do not necessarily represent those of their affiliated organizations, or those of the publisher, the editors and the reviewers. Any product that may be evaluated in this article, or claim that may be made by its manufacturer, is not guaranteed or endorsed by the publisher.
